# Different gait tasks distinguish immediate vs. long-term effects of concussion on balance control

**DOI:** 10.1186/1743-0003-6-25

**Published:** 2009-07-07

**Authors:** Robert D Catena, Paul van Donkelaar, Li-Shan Chou

**Affiliations:** 1 Motion Analysis Laboratory, Department of Human Physiology, University of Oregon, Eugene, Oregon 97403-1240, USA

## Abstract

The purpose of this study was to longitudinally compare the sensitivity of previously documented paradigms for measuring balance control during gait following a concussion. We hypothesized that gait with a concurrent cognitive task would be most sensitive to the effects of concussion on dynamic balance control. Individuals with concussion (n = 30) and matched controls (n = 30) performed a single task of level walking, attention divided walking, and an obstacle-crossing task at two heights. Testing occurred four times post-injury. Balance control during gait was assessed with whole-body center of mass and center of pressure motion. The single-task level walking task did not result in any significant differences in balance control between individuals with concussion and control subjects. Within 48 hours post-injury, individuals with concussion walked slower and allowed less motion of their center of mass in the sagittal plane when attention was divided during walking, but there were no group differences by day 6 for this task. Group differences in balance control during obstacle crossing was unremarkable during the first two testing sessions, but by day 14 individuals with concussion displayed less mediolateral motion of their center of mass. Attention divided gait is able to better distinguish gait adaptations immediately following a concussion, but obstacle crossing can be used further along in the recovery process to detect new gait adaptations.

## Background

Although concussive incidents rarely result in any patient-reported long-term symptoms [1], studies have found that symptoms may last longer than that reported by the patient; even long after a return to normal unrestricted activities [2-4]. Although the specific causes of repeated concussions is unclear, it is our contention that one contributing factor may be related to the well documented [2-5] long-term deficits in dynamic motor function, such as balance control during walking. The rate of concussion has been shown to increase within several months after the first concussion [6] and multiple concussions occurring with unresolved symptoms can lead to permanent brain damage or increased probability of fatality depending on the time interval between concussive episodes [7].

Neuropsychological testing following concussion has been well documented and is routinely performed at least in the sports setting [8,9]. Symptoms measured with neuropsychological tests are often reported normal after 14 days post-injury. However, findings of motor dysfunction, gait imbalance and attentional deficits during motor/cognitive dual-task tests have contradicted this quick (within two weeks) return to normal functioning. A group of predominately mild traumatic brain injury (mTBI) subjects were reported displaying deficits in finger tapping up to a year post-injury [3]. Children with mTBI displayed balance deficits up to 12 weeks post-injury [4]. Severe TBI subjects have shown balance control deficits while performing obstacle crossing approximately a year after injury [5]. College-aged adults with concussion showed decreased dynamic balance control during an attention dividing task a month post-injury [2].

Recently, tests of balance control during an attention dividing task have been proposed as an alternative method for assessing college-aged individuals following concussion [10,11]. When compared to other gait scenarios, gait with a secondary question and answer task was found to better differentiate changes in balance control between patient and control populations within two days post-injury. While obstacle crossing was deemed ineffective in distinguishing individuals with concussion immediately following concussion in the same study [10], others have previously used obstacle crossing tasks to successfully detect balance control deficit in more severely injured subjects months after the injury [5,12].

To our knowledge, a longitudinal examination of balance control comparing two balance perturbing gait tasks (divided attention walking vs. obstacle crossing) has not been performed with individuals suffering from concussion. Such information would uncover dynamic balance deficits following concussion during both tasks, while simultaneously identifying the most sensitive test to such balance deficits. If deficits do exist and tests for such deficits are clinically implemented then patients with concussion may have a more exact timeframe to limit motor activities and avoid subsequent concussion.

The purpose of this study was to examine dynamic balance control over a one month period, using gait protocols that have been previously reported separately, to determine a gait scenario that can effectively detect changes in balance control of individuals with concussion and can be used to track recovery. We hypothesized that a concurrent cognitive task would most effectively accomplish both of these purposes based on previous reports.

## Methods

### Subjects

Thirty subjects with concussion (mTBI) were referred to testing by the student health center or athletic team physicians/trainers of the university campus. MTBIs (14 females/16 males; age = 21.5 ± 3.3 years; mass = 83.2 ± 24.7 kg; height = 176.7 ± 10.8 cm) were diagnosed with grade II concussions as defined by the American Academy of Neurology Practice Parameters [13], which entails symptoms lasting longer than fifteen minutes, but no loss of consciousness. Exclusion criteria included preexisting abnormalities in gait or cognition. Sixteen mTBI participants had a previous concussion a year or more prior to testing but none indicated any lingering symptoms. Subjects in this study ranged from non-athletic to intercollegiate athletes and all were still participating in their particular activity at the time of injury at either the college or professional level, or have since graduated and are no longer active.

Thirty control subjects were matched by gender, age (21.7 ± 3.1 years), mass (82.6 ± 23.9 kg), height (175.9 ± 10.4 cm), level of education and athletic participation. Exclusion criteria were the same as that for mTBI subjects, in addition to exhibiting common symptoms of concussion described by Collins et al [14]. Ten controls had a previous concussion more than 1.5 years prior to this study, but none complained of any lingering effects and were functioning normally in society and academics. There was no statistical significance in balance measures between control individuals that did and did not have a previous concussion (greatest *p *= 0.460). Approval for the use of human subjects was granted prior to testing by the university Institutional Review Board. Written and verbal instructions of testing procedures were provided, and written consent was obtained from each subject prior to testing.

### Apparatus

Twenty-nine retroreflective markers were attached to anatomical landmarks [15]. Three dimensional marker trajectories were collected with an eight camera motion tracking system (MotionAnalysis Corp.) at 60 Hz. The cameras were positioned surrounding an eight-meter walkway. Ground reaction forces and moments were collected at 960 Hz with two in-ground force plates (Advanced Mechanical Technologies Inc.). A PVC pipe (1/2" diameter, 1.3 m length) set atop two adjustable uprights between the two force plates was used as an obstacle.

### Protocol

The first testing (day 2) for mTBI subjects occurred within 48 hours post-injury (35.8 ± 13.1 hours). Data collection started with a single-task level walking session (LEVEL). Subjects were asked to walk at a comfortable self-selected pace while barefoot. Several practice trials were allowed so that subjects could become accustomed to walking with the marker set.

Shorter and taller obstacle crossings were then performed in two blocks. During short obstacle crossing (OBS) the obstacle was set to a 4 cm height. During tall obstacle crossing (OBT) the obstacle was set at 10% of the subject's body height. The final trial block was a divided attention task (Q&A). Subjects performed unobstructed gait while continuously responding to a question posed at the beginning of each trial. Questions included: spelling a common five-letter word in reverse, continuous subtraction by a certain number, and reciting the months of the year in reverse order [2,10,11,16,17]. At the beginning of each trial the subject was given the specific task for that trial (e.g. count backwards by sevens starting at ninety-three). The subject then started walking and answering at the same time and stopped answering at the end of the walkway. Each testing session lasted about 30 minutes with breaks between trial blocks. MTBI subjects performed the same set of tasks at the approximate 6^th ^day, 14^th ^day and 28^th ^day post-injury. Controls were tested at similar time intervals.

### Data processing

Marker trajectories were filtered with a low-pass fourth order Butterworth filter at a cutoff frequency of 8 Hz. Marker position data were used to locate the segmental center of mass (CoM) of a thirteen-link model including: head, trunk, two upper arms, two lower arms, pelvis, two thighs, two shanks, and two feet, based on Dempster's anthropometric data [18]. A weighted sum method was used to calculate the whole body CoM during each time point. CoM motion data were analyzed between the first heel strike on to the first force plate to the next heel strike of the same foot. CoM velocities were estimated with the use of Woltring's generalized cross-validated spline algorithm [19]. Center of pressure (CoP) data were calculated from force plate data.

A model of how balance is maintained through proper positioning of the CoM and momentum of the CoM over the base of support has been established as a measure of dynamic balance control [20,21]. In this study of walking balance control, CoM sagittal and coronal plane range of motion (AP ROM and ML ROM), and peak velocities in the anterior-posterior (AP V) and mediolateral directions (ML V) were identified. CoM data were synchronized with the CoP data to find the maximum horizontal separation distance between the CoM and CoP in both the sagittal plane (APmax) and coronal plane (MLmax). Data from three to five successful trials were averaged together for each group, day, and task to complete the statistical analyses.

Together the aforementioned variables allow us to examine two important aspects of dynamic balance control: a conservative adaptation to walking and the likelihood of imbalance during walking. Conservative adaptations include a stride time and step length decrease. These correspondingly reduce AP ROM, AP V and APmax. However, it is not necessary that these three variables are correlated. One can alter step length and stride time by varying amounts up or down, partially independent of each other. For this reason, we identified each of the AP variables for conservative adaptations. APmax correlates with a step length. AP V is a combination of stride length and stride time without consideration of subject height. AP ROM correlates with stride length and stride time with consideration of subject height. ML variables demonstrate the dynamic balance of an individual [5]. Specifically the MLmax variable corresponds with maximum step width. ML ROM corresponds with average step width. ML V has no intuitive relation with other common temporal-distance variables; however, this can be an important variable to describe imbalance from side to side [20]. Although the other variable do have a correlation to temporal-distance variables, the use of COM variables allows us to more directly and intuitively measure balance.

### Statistical analysis

Although not completely exclusive, the dependent variables were not analyzed with a MANVOA because they did not meet linearity criteria. Appropriate assumptions for mixed ANOVAs were analyzed and considered tenable. Upon these assumptions being met, a three-way (2 groups, 4 tasks, and 4 days) mixed model analysis with repeated measures (alpha = 0.05) was conducted using SAS 9.1 (SAS Institute Inc., Cary, NC). The data were analyzed following appropriate top-down methods (3-way interaction, 2-way interactions, main effects). Follow-up pairwise comparisons with adjustments for multiple comparisons were performed when statistical significance was determined in the mixed model. To account for multiple comparisons and avoid Type I error, alpha levels were set a priori at 0.0167 for pairwise comparisons based on recommendations about error rates relative to individual family size [22].

## Results

The results for sagittal plane balance control clearly indicate that individuals with concussion reduce their forward motion immediately after injury when having to perform a divided attention gait task. A three-way interaction in AP ROM (*p *= 0.0030) showed that participants with concussion had less sagittal plane CoM displacement than controls on day 2 during the Q&A task (*p *= 0.0143). A group-by-day interaction in AP V (*p *< 0.0001) showed that participants with concussion also significantly reduced their peak anterior CoM velocity on day 2 during the Q&A task (*p *= 0.0135; Table 1). APmax also showed a group-by-day interaction (*p *= 0.0187), however further analysis only determined a trend of participants with concussion allowing less separation between their CoM and CoP in the anterior direction on day 2 during the Q&A task (*p *= 0.0381). A summary of statistical results is presented in Table 2.

**Table 1:** Mean values (standard deviations) of COM variables.

**Dependent Variable**	**Task**	**Group**	**Time (days)**			
			
			**2**	**6**	**14**	**28**
AP V(m/s)	LEVEL	mTBI	1.393 (.141)	1.494 (.152)	1.517 (.152)	1.530 (.152)
		Cont.	1.416 (.164)	1.477 (.172)	1.478 (.158)	1.508 (.166)
	Q&A	mTBI	**1.245 (.163)**	1.382 (.179)	1.419 (.154)	1.436 (.174)
		Cont.	**1.326 (.172)**	1.405 (.186)	1.428 (.197)	1.445 (.197)
	OBS	mTBI	1.390 (.145)	1.470 (.157)	1.492 (.146)	1.505 (.154)
		Cont.	1.426 (.165)	1.484 (.187)	1.486 (.168)	1.497 (.175)
	OBT	mTBI	1.342 (.136)	1.435 (.159)	1.453 (.162)	1.458 (.161)
		Cont.	1.401 (.177)	1.465 (.183)	1.453 (.167)	1.477 (.183)

MLmax (m)	LEVEL	mTBI	0.080 (.025)	0.080 (.026)	0.078 (.021)	0.079 (.024)
		Cont.	0.076 (.017)	0.079 (.019)	0.078 (.023)	0.084 (.028)
	Q&A	mTBI	0.084 (.023)	0.082 (.024)	0.081 (.025)	0.077 (.021)
		Cont.	0.080 (.022)	0.080 (.019)	0.082 (.019)	0.086 (.028)
	OBS	mTBI	0.079 (.025)	0.075 (.019)	0.077 (.023)	**0.072 (.020)**
		Cont.	0.076 (.019)	0.076 (.018)	0.084 (.024)	**0.087 (.034)**
	OBT	mTBI	0.079 (.025)	0.076 (.023)	0.080 (.032)	**0.074 (.022)**
		Cont.	0.075 (.017)	0.078 (.024)	0.085 (.030)	**0.090 (.036)**

ML V (m/s)	LEVEL	mTBI	0.134 (.036)	0.132 (.035)	0.134 (.030)	0.132 (.030)
		Cont.	0.133 (.028)	0.140 (.031)	0.138 (.031)	0.135 (.031)
	Q&A	mTBI	0.148 (.036)	0.148 (.036)	0.145 (.033)	0.145 (.034)
		Cont.	0.149 (.032)	0.159 (.032)	0.150 (.030)	0.149 (.038)
	OBS	mTBI	0.144 (.034)	0.143 (.036)	**0.139 (.026)**	0.135 (.027)
		Cont.	0.146 (.035)	0.151 (.039)	**0.157 (.036)**	0.148 (.039)
	OBT	mTBI	0.157 (.029)	0.155 (.038)	0.147 (.029)	**0.148 (.029)**
		Cont.	0.156 (.036)	0.159 (.036)	0.164 (.038)	**0.168 (.043)**

The two group means in bold are significantly different from each other.

**Table 2:** P-values from statistical analyses conducted in this study.

Dependent variable	3-way interaction	2-way interactions			Main effects		
		Group*Day	Task*Day	Group*Task	Group	Task	Day
AP ROM	0.0030						
AP V	0.3425	< 0.0001	< 0.0001	0.4008			
APmax	0.1262	0.0187	< 0.0001	0.7161			
ML ROM	0.7628	0.1596	0.1292	0.8745	0.5969	< 0.0001	0.0027
ML V	0.0228						
MLmax	0.5858	< 0.0001	0.0125	0.6423			

Blank cells indicate the statistical analysis was not analyzed at this level because higher levels were significant.

The results for coronal plane balance control indicate that individuals with concussion initially are not different from controls, but they begin to use less coronal plane motion while crossing an obstacle two weeks after injury. A three-way interaction in ML V (*p *= 0.0228) showed that participants with concussion had significantly slower sideways peak velocities by day 14 for the shorter obstacle crossing task (*p *= 0.0143; Table 1) and by day 28 for the taller obstacle crossing task (*p *= 0.0128). A group-by-day interaction in MLmax (*p *< 0.0001) showed that mTBIs also reduced their CoM-CoP separation distance in the medial/lateral direction by day 28 for both obstacle crossing heights (OBS: *p *= 0.0006; OBT: *p *= 0.0018; Table 1). There were no significant group differences in ML ROM. A summary of statistical results is presented in Table 2.

## Discussion

The purpose of this research was to examine several different commonly used gait paradigms to determine which if any would most effectively distinguish balance control deficits following a concussion. The statistical analyses indicated that single task level walking was not able to effectively distinguish the two groups at any time point in the recovery process. Previous reports have consistently demonstrated a tendency for individuals with concussion to adopt a more conservative gait strategy by either walking slower and/or allowing less motion of the CoM in the sagittal plane immediately following the concussion [2,10,11,17]. Our current results showed a trend of this conservative gait strategy adopted during level walking immediately after the concussion. We believe that relatively minute changes in gait during level walking were indistinguishable when comparing so many tasks with relatively large differences between groups during other tasks. Previous analyses of gait have yielded some inconsistent results for coronal plane motion during single task level walking, some indicated group differences and some showing differences [2,10,11,17]. Current findings and inconsistencies in the literature may suggest that an analysis of single-task unobstructed gait can not adequately distinguish individuals with concussion and will not be able to consistently and accurately track their recovery.

Immediately following a concussion, level walking with a concurrent cognitive (Q&A) task was able to distinguish individuals with concussion from uninjured controls better than other gait tasks. Our results on day 2 during the Q&A task are in accordance with previously reported results that not only showed reduced gait velocity due to a concussion, but also reduced sagittal plane motion of the CoM to indicate a conservative gait adaptation to this task [2,10,11,17]. Center of mass trajectories have been previously described as providing insight specifically into dynamic balance control mechanisms [20,23]. By day 6 the Q&A task no longer detected any group differences. This suggests that the average individual with concussion had recovered enough from any attentional deficits they might have had following the concussion that balance control was no longer affected. This quick return to normal is in line with many neuropsychological findings [8,24]. The spatial orientation component of attention has also been reported to return to normal by five days post-injury, while the executive function component of attention still showed signs of deficit up to a month post-injury [25]. The combination of slower processing speed, deficits in the ability to spatially orient attention and deficits in switching attention between tasks have been used to describe the increased challenge that individual with concussion are subjected to in a dual-task walking situation [26]. The fact that only spatial orientation of attention improves by five days post-injury while other aspects of attention remain disabled up to a month post-injury [25] in combination with our results may indicate that either the remediation of any part of attention helps in performance during dual-task walking or that remediation of spatial orientation of attention is correlated with the recovery of other attention components that would be more likely to aid in Q&A task performance. While improved performance by 6 days post-injury is contradictory to a previous report that showed reduced sagittal CoM motion in gait even up to a month post-injury when attention was divided [2], a trend in our data may suggest similar results. The conflicting statistical significance could be an indication of the heterogeneity in concussive symptoms between subjects, further supporting our recommendation for individual motor/attention tests prior to a return to activity.

The two groups displayed no statistical differences in CoM motion when performing obstacle crossing in the first week of testing. Similar findings have also concluded that obstacle crossing was less effective at distinguishing individuals with mild concussion immediately following injury [10]. However, our longitudinal analysis of obstacle crossing revealed interesting findings at the two- and four-week testing sessions. By day 14, individuals with concussion showed the first signs of statistically different mediolateral CoM motion. They had reduced mediolateral peak velocities of the CoM by day 14 and also reduced mediolateral separation of the CoM and CoP on day 28. Both of these indicate a conservative control of mediolateral balance based on a distance-velocity model of the CoM with respect to the base of support [20] in individuals with concussion. Others have also suggested eventual conservative balance control during obstacle crossing [12]. By reducing CoM motion in the coronal plane, sideways imbalance might be better avoided [11].

There are several possible reasons as to why mediolateral control mechanisms are adopted only by 14 days after concussion. Each reason implies that AP and ML control are a least partially uncorrelated, to which other work attests [5,15,27,28]. The first possibility is a reacquisition of mediolateral balance control. This hypothesis implies group differences in mediolateral balance control prior to day 14. The data however indicated that both groups had similar frontal plane CoM motion during the first two testing sessions. Nevertheless, similar values might not necessarily indicate similar performance if one group (mTBI) was required to apply greater effort (as has been previously suggested for cognitive test performance by individuals with concussion [29]) in controlling mediolateral balance during walking, while the control group accomplished the same task with less effort. Examining obstacle crossing with simultaneous Q&A performance might be able to shed light on this premise.

The second possibility is that individuals with concussion felt no need for greater demand in mediolateral balance while performing obstacle crossing prior to day 14. Poor decision making [30] and a lack of full task/environmental awareness [31] immediately following the concussion may have led to a false sense of ability and security during obstacle crossing. Only after this commonly reported "mental fogginess" subsided did individuals with concussion understand the importance of a safe obstacle crossing strategy when taking into account their reduced strength and coordination [14] needed to arrest the body during a possible trip and desire to avoid re-injury.

The third possibility is that confining mediolateral CoM motion could be due to increased comfort performing this particular task. Anxiety for several weeks following mild brain injury has been documented [32]. Gradually increasing comfort with their ability to safely cross over the obstacle without obstacle contact may allow the individuals with concussion to focus more attention on medial/lateral balance control. Further analyses of obstacle crossing parameters may be used to test these hypotheses.

A major limitation to our study is that control subjects did not perform similarly each day. Although not statistically significant, control subjects also displayed a change in gait performance over time indicating a decrease in performance anxiety in each subsequent testing session. However, all subjects were tested in similar conditions, so any change in performance due to anxiety would be expected in both groups rather than just one. This indicates that normal changes in performance due to comfort with the testing protocol are also imbedded in the longitudinal curves for subjects with concussion. Another limitation is the inclusion of individuals with previous concussions within both groups. This was unavoidable given the limited sample size in the group with concussion and the matching criteria in the control group. We however believe that not allowing individuals to participate if they had a concussion within a year prior is sufficient in excluding individuals still suffering from previous symptoms since there are no reports of symptoms of a mild (no loss of consciousness) concussion lasting longer than one year.

## Conclusion

Our findings indicated that a divided attention task performed during unobstructed gait was only able to better distinguish conservative gait adaptations immediately following a concussion. By day 6, attention had seemed to recover to the point at which the attention dividing task was no longer effective in perturbing balance control in individuals with concussion. By day 14, a more conservative control of mediolateral CoM motion was observed in the group with concussion during obstacle crossing. An attention dividing task and obstacle crossing task seem to detect changes in gait adaptations at different times in the recovery process. The inclusion of at least an obstacle crossing task may be advantageous in clinically detecting a recovery of functional balance control during gait based on data from this study. This information may someday lead to the regular inclusion of appropriate and clinically executable dynamic balance control tests after concussion. However, a longer longitudinal study where obstacle crossing returns to normal is recommended to determine that functional balance control has fully recovered. Finally, this work clearly points out the importance of further investigation of the complex issue of balance control following concussion.

## Competing interests

The authors declare that they have no competing interests.

## Authors' contributions

RDC and LSC designed the concept of study; RDC drafted the manuscript; PVD and LCS edited and revised the manuscript. All authors read and approved the manuscript.
